# Modification of Flight and Locomotion Performances, Respiratory Metabolism, and Transcriptome Expression in the Lady Beetle *Harmonia axyridis* through Sublethal Pesticide Exposure

**DOI:** 10.3389/fphys.2017.00033

**Published:** 2017-02-10

**Authors:** Da Xiao, Xiaoling Tan, Wenjuan Wang, Fan Zhang, Nicolas Desneux, Su Wang

**Affiliations:** ^1^Institute of Plant and Environment Protection, Beijing Academy of Agriculture and Forestry SciencesBeijing, China; ^2^Institute of Plant Protection, Chinese Academy of Plant ProtectionBeijing, China; ^3^Institut National de la Recherche Agronomique (French National Institute for Agricultural Research), Univ. Nice Sophia Antipolis, Centre National de la Recherche Scientifique, UMR 1355-7254 Institute Sophia AgrobiotechNice, France

**Keywords:** mobility, behavior, sublethal effect, beta-cypermethrin

## Abstract

Biological control is usually used in combination with chemical control for practical agricultural applications. Thus, the influence of insecticides on the natural predators used for biological control should be investigated for integrated pest management. The ladybird *Harmonia axyridis* is an effective predator on aphids and coccids. Beta-cypermethrin is a broad-spectrum insecticide used worldwide for controlling insect pests. *H. axyridis* is becoming increasingly threatened by this insecticide. Here, we investigated the effect of a sublethal dose of beta-cypermethrin on flight, locomotion, respiration, and detoxification system of *H. axyridis*. After exposure to beta-cypermethrin, succinic female adults flew more times, longer distances, and during longer time periods. Exposure to a sublethal dose of beta-cypermethrin also promoted an increase in walking rate, walking distance, walking duration, and also an increase in respiratory quotient and respiratory rate. To investigate the effects of beta-cypermethrin on *H. axyridis* detoxification system, we analyzed the transcriptome of *H. axyridis* adults, focusing on genes related to detoxification systems. *De novo* assembly generated 65,509 unigenes with a mean length of 799 bp. From these genes, 26,020 unigenes (40.91% of all unigenes) exhibited clear homology to known genes in the NCBI non-redundant database. In addition, 10,402 unigenes were annotated in the Cluster of Orthologous Groups database, 12,088 unigenes were assigned to the Gene Ontology database and 12,269 unigenes were in the Kyoto Encyclopedia of Genes and Genome (KEGG) database. Exposure to beta-cypermethrin had significant effects on the transcriptome profile of *H. axyridis* adult. Based on uniquely mapped reads, 3,296 unigenes were differentially expressed, 868 unigenes were up-regulated and 2,248 unigenes were down-regulated. We identified differentially-expressed unigenes related to general detoxification systems in *H. axyridis*. This assembled, annotated transcriptome provides a valuable genomic resource for further understanding the molecular basis of detoxification mechanisms in *H. axyridis*.

## Introduction

Predatory ladybirds are key biocontrol agents either through inundatively, inoculatively, or conservation methods, notably for possible in combination with chemical, botanical, and microbial for suppressing arthropod pests in agricultural integrated pest management (IPM) system (Galvan et al., 2005; Lu et al., 2012). Predatory ladybirds play a key role in pest control, e.g., after pesticide application to prevent resurgence of pest and limit their populations below economic threshold values. Various IPM methods have been developed to reduce applications of pesticides and to prevent negative side effects on biocontrol agents (Candolfi et al., 2001). However, these biocontrol agents could still be affected by the side-effects of pesticides, notably their residues (Desneux et al., 2004a; 2006a,b; Liu and Stansly, 2004; He et al., 2012; Yu et al., 2014; and see Desneux et al., 2007 for a through review of the topic).

Numerous studies have reported that sublethal doses of pesticides may impact arthropods (Liang et al., 2012; He et al., 2013; Pan et al., 2014; Chen et al., 2015; Fekri et al., 2016; Guedes et al., 2016) and notably natural enemies such as coccinellids (Desneux et al., 2007). Studies showed that various aspects of the biology of coccinellids may be altered by pesticides, including development (Yu et al., 2014), prey consumption (Wang and Shen, 2002; Singh et al., 2004; He et al., 2012), reproduction (Galvan et al., 2005; Rahmani and Bandani, 2013; Xiao et al., 2016), and metabolic mechanisms related to detoxification (Zhang et al., 2012; Tang et al., 2014). Evaluating the side-effects of sublethal doses of pesticides may help depicting possible adaptations of predatory insects to sublethal toxic pressures; it may also provide more information to improve the application of biological control agents in combination with sprayed pesticides in agricultural IPM systems.

Ladybirds are known as a group showing strong capacity for pest control. This high performance is related to their high mobility and their strong capacity to forage for prey. These abilities are often measured as flight and locomotion performances. Optimal flight ability may allow ladybirds to disperse over wider areas and to easily switch colonization positions (Majerus and Kearns, 1989). Rapid movement also could contribute to ladybird efficiency for prey hunting. Ladybird flight and locomotion are impacted by various environmental factors, e.g., temperature, light, floral habitats, and diets (Copp, 1983; Tan et al., 2014). Because of their high plasticity leading to adaptation, some ladybird species (e.g., *H. axyridis*) show advantages for new habitats, allowing ladybirds to compete with native species (Brown et al., 2011).

Environmental toxicants, such as pesticides, have been regarded as one of the key driving forces that accelerate genetic variation in insects. However, little is known about the responses of ladybirds to toxicants, and it is unknown whether exposure to sublethal doses of toxicants modifies ladybird flight and locomotion performances and/or metabolism.

The analysis of gene expression at the transcriptome level is a powerful tool to understand the effects of extrinsic environmental pressures on the molecular mechanisms that respond to adaptations. Some reports indicate that exposure to sublethal doses of pesticides may affect transcriptional regulation, inducing the up-regulation of genes related to detoxification, digestion, nutrition, metabolism (Zhang et al., 2012). There is scarce information about transcriptional changes related to flight, locomotion, and metabolism in ladybirds or in other predatory insects.

The harlequin ladybird, *H. axyridis* is known worldwide as a highly efficient biological control agent in its native Asia (Koch, 2003). It is also known as an unstoppable invader in regions where it was introduced (Brown et al., 2008; Van Lenteren et al., 2008). Several studies have shown that *H. axyridis* can efficiently cope with environmental changes and out-compete the native predatory insects in regions where it has been introduced (Vilcinskas et al., 2013; Mirande et al., 2015).

Both field and laboratory tests have shown that *H. axyridis* may suffer from exposure to various types of chemical, botanical, or microbial pesticides (Vincent et al., 2000). However, *H. axyridis* has many biological and physiological features that are adaptable. For instance, we found that *H. axyridis* treated with a sublethal dose of beta-cypermethrin developed faster and showed significantly increased fertility (Xiao et al., 2016). There are no reports analyzing changes in flight and locomotion performances of *H. axyridis* exposed to sublethal doses of pesticides. Flight and locomotion performances are important features for insect populations to disperse, migrate, overwinter, colonize new regions, and hunt prey.

In the present study, we analyzed flight and locomotion performances, as well as the respiratory metabolism of *H. axyridis* adults exposed to a sublethal dose of beta-cypermethrin. Adult females of *H. axyridis* (of succinic phenotype i.e., non-melanic phenotype) were selected in our study, because different polymorphic adult types of *H. axyridis* (distinguished by different elytra patterns) may exhibit different responses to the environmental changes. Differential responses may be especially important for migration during winter and overwinter aggregation (Wang et al., 2011). Furthermore, we investigated the metabolism and detoxification, as possible physiological adaptations to exposure to sublethal doses of insecticide. Differentially-expressed genes (DEGs) were compared between exposed and non-exposed insects by transcriptome sequencing.

## Materials and methods

### Insects

A total of 416 adults of the ladybird *H. axyridis* (146 females and 270 males) were collected from an alfalfa field in the campus of Beijing Academy of Agricultural and Forestry Sciences (BAAFS), Beijing, China. The ladybirds were transported to the Institute of Plant and Environment Protection of BAAFS, and maintained in culture cages (45.0 × 50.0 × 50.0 cm) made of aluminum frames and 80 mesh plastic nets. Ladybirds were maintained at a density of 30 pairs per cage. Newly-laid eggs were moved to a new plastic container (30.0 × 40.0 × 30.0 cm) until the first instar larvae emerged. Larvae (30 per cage) were kept in culturing cages with folded paper tape as filler to avoid cannibalism. Newly-eclosed adults were isolated in different plastic containers and separated by sex (the sex of the adults was determined by the color of their mouthpart top: Black/dark = female and white/light = male) and polymorphism (i.e., melanic or succinic phenotype).

Old nymphs of the aphid *Myzus persicae* were provided daily as food (*Ad libitum*). Three-day-old virgin succinic females of similar body weight were collected for the experiments. Ladybirds were reared in regulated environments with the following characteristics: T = 25°C, relative humidity = 65%, photoperiod = 16 h light and 8 h dark, illumination intensity = 1,200 lux. All the conditions were managed by an automatic environment regulation system (L-100, Suntech, Beijing, China).

### Analysis of flight, locomotion, and respiratory metabolism

#### Exposure of *H. axyridis* to a sublethal dose of beta-cypermethrin

The LC_5_ of beta-cypermethrin for *H. axyridis* was 3.158 mg L^−1^ as it was estimated in a sister study by Xiao et al. (2016). The ladybirds were exposed to the insecticide using the method of exposure to pesticide residues in glass tubes (see Desneux et al., 2004a,b,c). An aliquot of 820 μL of the insecticide solution in acetone was placed in each glass tube (14.5 cm long with a diameter of 1.5 cm). The ladybird exposed to acetone only was used as control. These tubes were immediately rotated using a micro-rotator (American Wheaton Company) until their insides had been evenly coated with the insecticide residue. The ladybirds were transferred into the tubes for 24 h, then used in the following experiments.

#### Flight mill test

**Figure 1:**
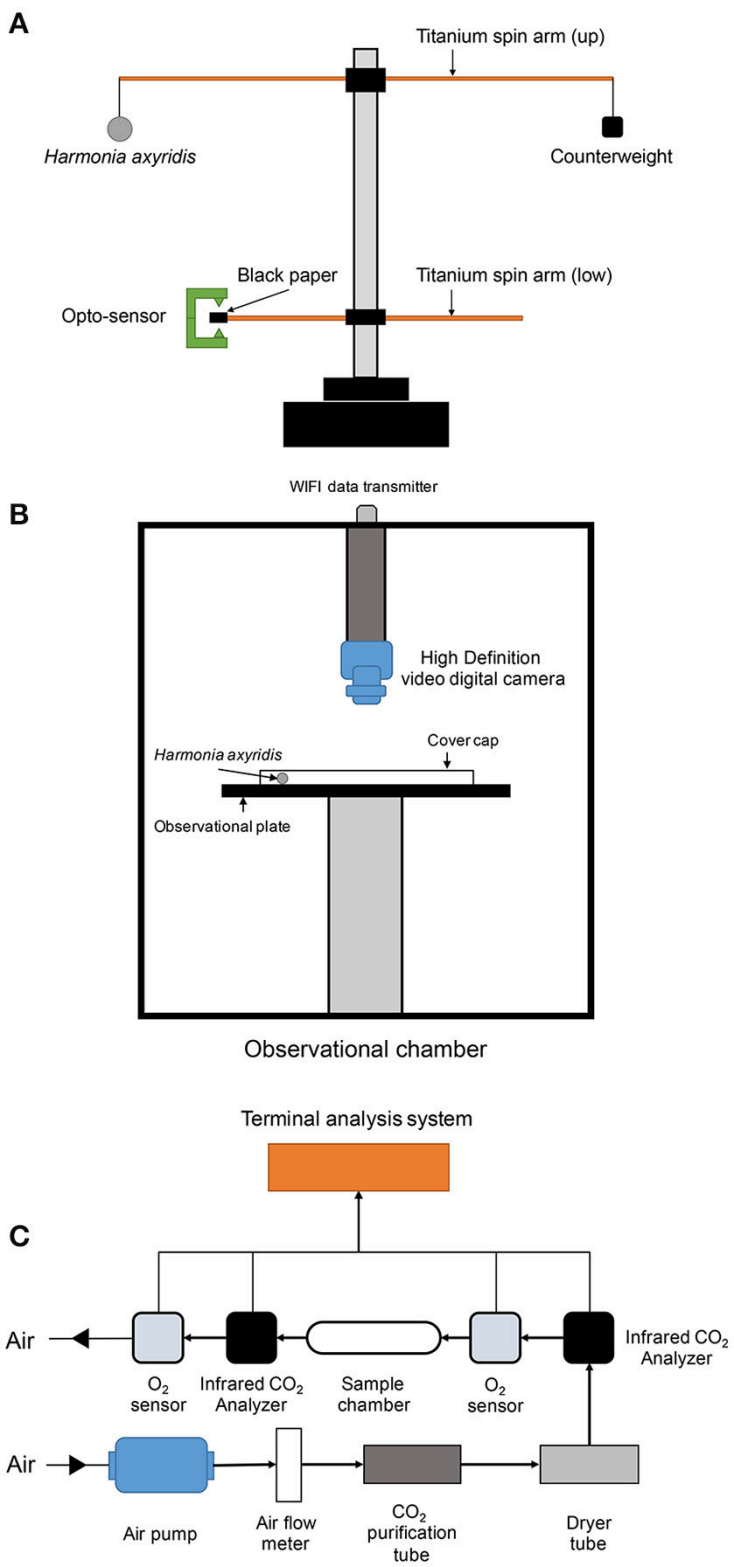
**Setup of flight mill apparatus (A)**, flight locomotion behavior-monitoring chamber **(B)**, and respiration detector **(C)**.

A 16-channels flight mill system (DL1100, Jiaduo, Henan, China) (Figure 1A) was used and adjusted for testing the flight ability of *H. axyridis*. The 16-channels flight mill system allowed us to measure the flight performance of 16 insects simultaneously. For the analysis of flight performance, 3-days-old virgin adults (succinic female) of *H. axyridis*, exposed or not to beta-cypermethrin, were starved for 6 h. A plastic thread (diameter = 0.2 mm, length = 2.0 cm) was used to link the ladybird to the top of the longer side of the titanium spin arm (Figure 1A). The thread was adhered to the tergum of ladybird adults without influencing their flight activity.

We adjusted the counterweight (positioned on the top of the shorter side of titanium spin arm) to keep whole spin arms in balance. The computer program (ST-moni 1.0, Jiaduo, Henan, China), receiving the electric signals from an opto-sensor (Figure 1A), was turned on once the ladybirds were well in place. Once the ladybirds started to flight, the lower spin arm rotated in a synchronized way with the upper spin arm. When the black paper piece on the lower spin arm crossed the opto-sensor, as the arms were spinning, the computer program read and recorded the signal automatically. The program could record and calculate data for a selected observation period. Calculations made were as follow: Total flight frequency (number of times flight was initiated), accumulative flight duration and distance, and average flight speed. The flight mill test was conducted for 3 h (180 min) inside a rearing chamber, which was environmentally regulated as described above to keep the conditions constant. We repeated the test for 3 groups including 16 ladybirds in each group (a total 48 ladybirds were tested).

#### Locomotion test

We utilized a CASO L-1 Arthropod Locomotion Tracking System (Camsor, Sheffield, UK) (Figure 1B) to compare the locomotion performance of *H. axyridis* adults. The locomotion system consisted of an observation chamber and a computer program for analysis (Image LE 1.2 ver.). The observation chamber was environmentally regulated to automatically maintain stable conditions. An observation plate (45.0 cm of diameter) of black color was set in the middle of the chamber. Thirty centimeters above the plate, a high definition digital video camera was set for monitoring and recording the locomotion activities of the insects. Succinic female adults, which were (or were not) exposed to beta-cypermethrin, were individually placed on the observation plate surface, which was then covered by a transparent glass cylindrical cap (height = 2.0 cm). The video recording started once the ladybirds had been kept for 10 min inside the chamber. Using the software for data analysis, we interpreted the data as: Walking frequency (the ladybird started moving and maintained the movement over 1 s), accumulative walking duration and distance, average walking speed, and direction change frequency (the ladybird direction changed over 45°). Each test was repeated 40 times for pesticide-exposed and control *H. axyridis*.

#### Respiratory metabolism analysis

Respiratory metabolism of *H. axyridis* adults were measured using an insect respiratory measurement system. The system consisted of a CO_2_ infrared analyzer (Testo535, Munich, Germany), an InPro-6000 model gas O_2_ sensor (Mettler-Toled, Switzerland), an air pump (SP200EC-LC, Schwarzer-Precision, Germany), an airflow meter, a CO_2_ purification tube, a drying tube, a glass sample chamber (diameter = 4.0 cm and height = 15.0 cm), a data collector (LabPro V, Vernier, USA), and a temperature sensor (TMP-112, Texas Instrument, USA) (Figure 1C). A terminal analysis program (Sable T, Sable system, Denmark) calculated the average respiratory quotient and respiratory rate directly from the raw data. Once a ladybird was placed into the glass sample chamber, the air pump was turned on and the airflow adjusted to 0.1 L/min. The environmental conditions were regulated by the environmental condition management system as described above. All the sensors and data collectors were kept in recording status for 30 min, and then the respiratory quotient and respiratory rate of insects were calculated.

### cDNA library construction and illumina sequencing

Two libraries were constructed, one for ladybirds that were (and one for ladybirds that were not) exposed to beta-cypermethrin for 24 h. Total RNA was isolated using TRIzol reagent (Invitrogen, Carlsbad CA, USA) according to the manufacturer's instructions. RNA was then treated with DNase. RNA sample concentration and integrity were determined using a 2100 Bioanalyzer (Agilent Technologies). Poly A-containing mRNAs were enriched using oligo (dT) magnetic beads and then fragmented using RNA Fragmented Reagent. First- and second-strand cDNA was synthesized, purified, and end-repaired. Single nucleotides were added, adapters ligated, and the ligated products purified. Finally, cDNA templates were enriched by PCR amplification. The quality of the library was evaluated and the amount of cDNA quantified using an Agilent 2100 Bioanalyzer and a ABI StepOnePlus Real-time PCR system, respectively. The cDNA was then sequenced at 90 bp using the Illumina HiSeqTM 2000 platform at the Beijing Genomics Institute (BGI, Shenzhen, China).

### Transcriptome analysis

We first filtered out the sequencing adaptors, the unknown nucleotides larger than 5%, and the low quality reads. The resulting clean reads were assembled using Trinity (Grabherr et al., 2011). The resulting sequences from Trinity assembly were considered as unigenes. For annotation, unigenes were aligned by BLASTx, using an E-value cut-off of 10^−5^, against the NCBI non-redundant Swiss-Prot Kyoto Encyclopedia of Genes and Genome (KEGG, http://www.genome.jp/kegg/), and against the Cluster of Orthologous Groups (www.ncbi.nlm.nih.gov/COG) protein database. Gene Ontology of unigenes was analyzed using the Blast2Go software (Conesa et al., 2005). Gene ontology functional classification of all unigenes was performed using the WEGO software (Ye et al., 2006). In addition, unigenes without homology to these databases were forecast for their translation direction and open reading frames (ORF) using the ESTScan software (Iseli et al., 1999).

### Differentially-expressed gene analysis

The relative abundance of transcripts of *H. axyridis* that were (or were not) exposed to beta-cypermethrin was expressed as fragment per kilobase per million fragment, according to Mortazavi et al. (2008) Differentially-expressed genes between exposed and un-exposed *H. axyridis* were identified on the basis of the rigorous algorithm false discovery rate ≤ 0.001, and absolute value of log_2_ Ratio ≥ 1. Differentially-expressed genes were then subjected to gene ontology enrichment analysis and KEGG pathway enrichment analysis. For gene ontology enrichment analysis, the calculated *P*-value from the hypergeometric test underwent Bonferroni Correction, and the gene ontology terms with the corrected *P*-value ≤ 0.05 were significantly enriched in all differentially-expressed genes. For pathway enrichment analysis, pathways with a *Q* value ≤ 0.05 after multiple testing correction were significantly enriched in all differentially-expressed genes.

### Statistical analysis

The data obtained from the flight, locomotion and respiration tests were analyzed using *t*-test to separate the means between control and treatment (using ProStat software, Poly Software International).

## Results

### Comparison of flight, locomotion performances, and respiratory metabolism

**Figure 2:**
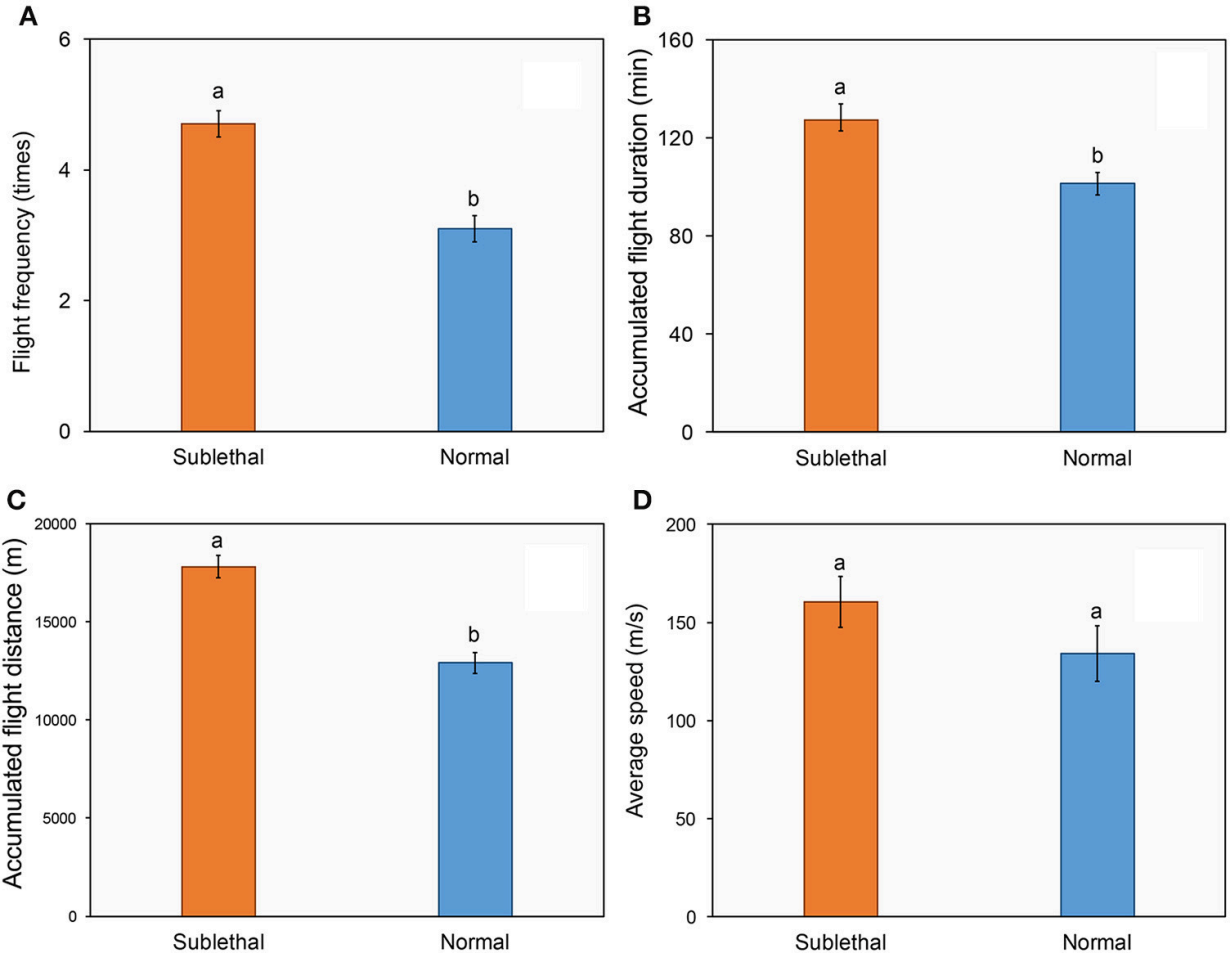
**Effect of a sublethal dose of beta-cypermethrin on the fight frequency (A)**, flight duration **(B)**, flight distance **(C)**, and flight speed **(D)** of *Harmonia axyridis*. The data are presented as the mean and standard errors of three replicates. Different letter above the standard error bar indicated significantly differences based on Testing following by *t*-test (*p* < 0.05).

Figures 2A–D shows the flight performance of succinic female adults of *H. axyridis* that were (or were not) exposed to a sublethal dose of beta-cypermethrin. The ladybirds that were exposed to beta-cypermethrin showed significantly higher flight frequency (Figure 2A, *t* = 5.13, *df* = 90, *P* < 0.01), longer accumulated flight duration (Figure 2B, *t* = 3.17, *df* = 89, *P* < 0.01), and longer accumulated flight distance (Figure 2C, *t* = 6.13, *df* = 90, *P* < 0.01) as compared with their respective control. By contrast, there were no significant differences of average flight speed between the (Figure 2D, *t* = 1.94, *df* = 88, *P* = 0.57) exposed and un-exposed ladybirds.

**Figure 3:**
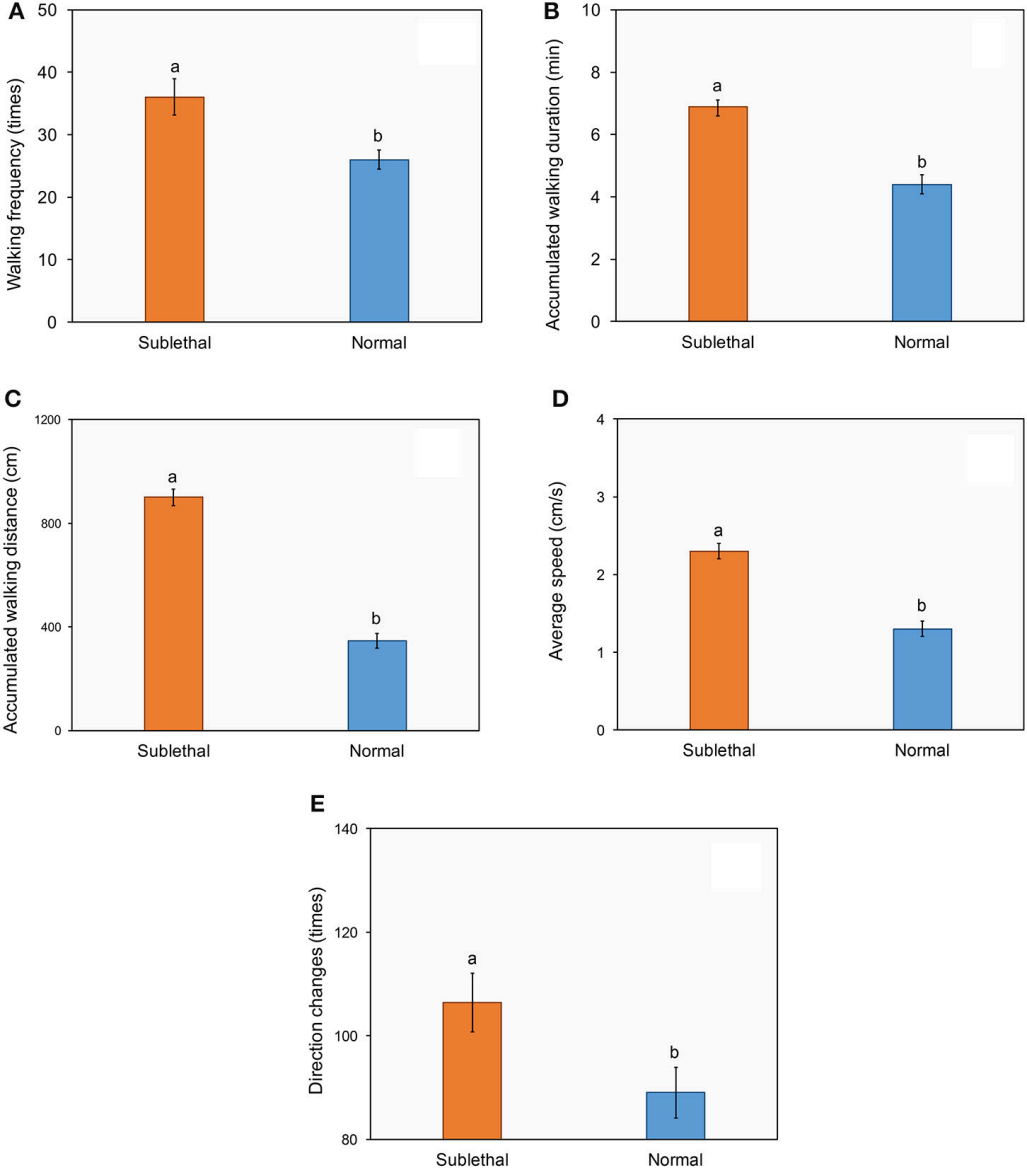
**Effect of a sublethal dose of beta-cypermethrin on the walking frequency (A)**, walking duration **(B)**, walking distance **(C)**, walking speed **(D)**, and direction change **(E)** of *Harmonia axyridis*. The data are presented as the mean and standard errors of three replicates. Different letter above the standard error bar indicated significantly differences based on Testing following by *t*-test (*p* < 0.05).

Similarly, ladybirds exposed to beta-cypermethrin showed significantly higher walking frequency (Figure 3A, *t* = 3.1, *df* = 58, *P* < 0.01), longer accumulated walking duration (Figure 3B, *t* = 6.87, *df* = 78, *P* < 0.01), longer accumulated walking distance (Figure 3C, *t* = 8.06, *df* = 66, *P* < 0.01), higher average walking speed (Figure 3D, *t* = 6.67, *df* = 78, *P* < 0.01) and a higher frequency of direction change (Figure 3E, *t* = 2.3, *df* = 78, *P* < 0.01) as compared with control.

**Figure 4:**
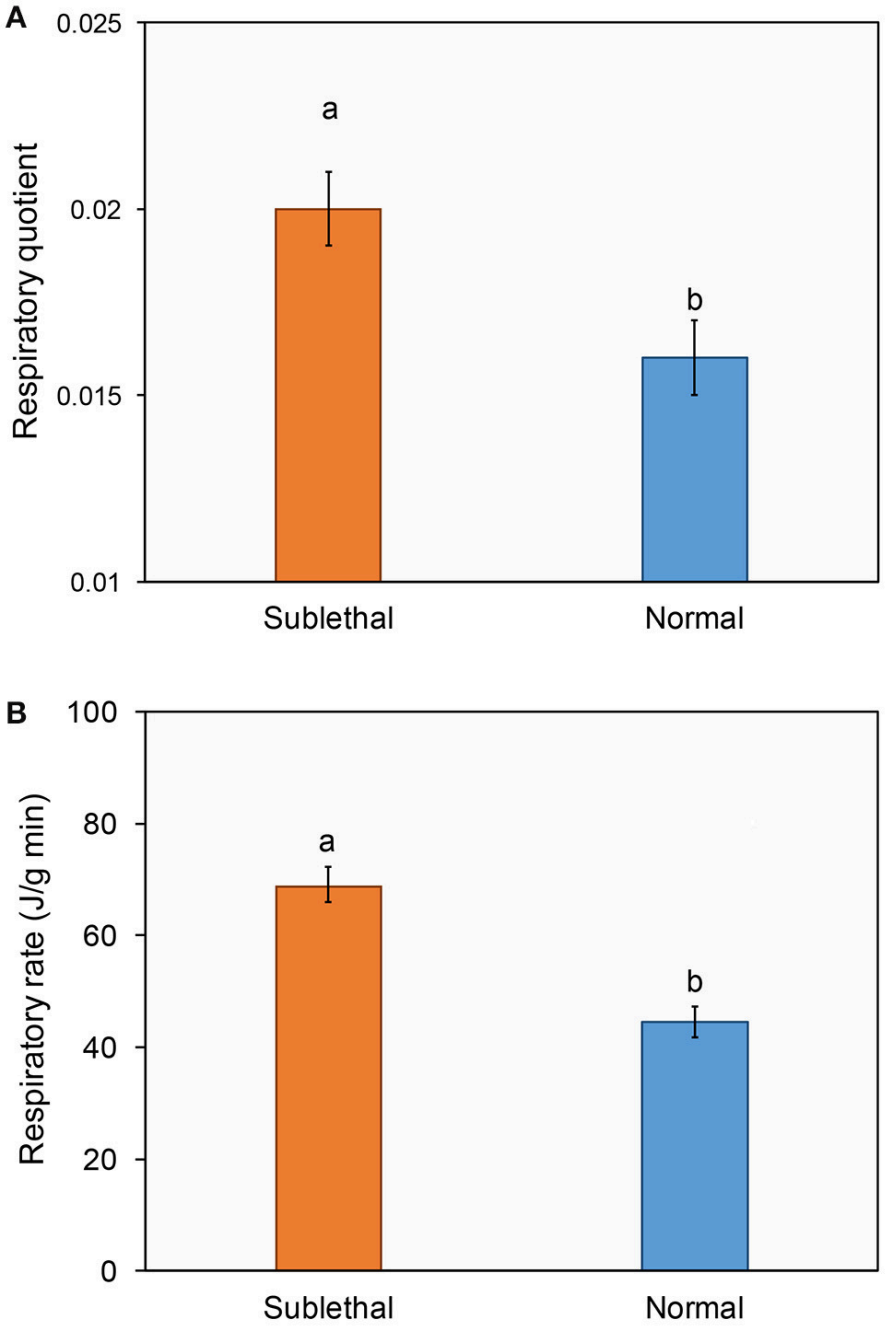
**Effect of a sublethal dose of beta-cypermethrin on the respiratory quotient (A)** and respiratory rate **(B)** of *Harmonia axyridis*. The data are presented as the mean and standard errors of three replicates. Different letter above the standard error bar indicated significantly differences based on Testing following by *t*-test (*p* < 0.05).

In addition, ladybirds exposed to beta-cypermethrin had a significantly higher respiratory quotient (Figure 4A, *t* = 2.23, *df* = 78, *P* = 0.014) and respiratory rate (Figure 4B, *t* = 5.48, *df* = 78, *P* < 0.01) as compared with their respective control.

### Illumina gene sequencing and *De Novo* gene assembly

RNA-seq analysis generated approximately a million paired-end reads from the exposed and un-exposed ladybirds. To obtain a comprehensive transcriptional profile of *H. axyridis*, the total clean reads from gene libraries of exposed and un-exposed ladybirds were combined. *De novo* assembly produced contigs with a mean length of 59.01 bp. These contigs were further assembled into 65,509 unigenes with an average size of 799.57 bp. There were 7,471 unigenes (14.96%) over 1000 bp in length (Figure S1). The N50 length of the contigs and unigenes was 704 and 795 bp, respectively.

### Functional gene annotation and classification

For functional annotation, all unigenes were aligned to the Genbank protein database with a cut-off E-value of 10^−5^ using BLASTx. Using this approach, 39.99% of all unigenes (26,020 unigenes) were above the cut-off value. The E-value distribution of the top hits in the non-redundant protein database showed that 41.9% of the unigenes (11,182 unigenes) had significant matches (< 1.0E-45); 58.1% of the matched unigenes had an E-value that ranged from 1.0E-5 to 1.0E-45. As for species distribution, most of the unigene sequences matched best to proteins from the red flour beetle (*Tribolium castaneum*; 37.44% of the unigenes), followed by the mountain pine beetle (*Dendroctonus ponderosae*; 5.23% of the unigenes), the pea aphid (*Acyrthosiphon pisum*; 1.32% of the unigenes) and the Asian citrus psyllid (*Diaphorina citri*; 1.29% of the unigenes) (Figure S2).

Gene ontology analysis was used to identify the potential function of the predicted proteins. A total of 12,088 unigenes were annotated and assigned to gene ontology terms. The most abundant groups were categorized as follows: Biological process, cellular component, and molecular function. In the biological process category (22,776 unigenes), the most abundant unigene groups were cellular process (5,297 unigenes), and metabolic process (5,100 unigenes). In the cellular component category (10,791 unigenes), the most abundant unigene group was cell part (3,719 unigenes). In the molecular function category (14,781 unigenes), the most abundant unigene groups were catalytic activity (6,588 unigenes) and binding (5,791 unigenes) (Figure S3). These results highlighted the importance of cell communication, metabolic activities, cellular structure, and molecular function in the life-cycle of *H. axyridis*.

Moreover, to further predict putative protein functions, cluster of orthologous groups analysis were performed. A total 14,258 unigenes were annotated and were classified in 25 categories (Figure S4). Among these 25 cluster of orthologous groups categories, the cluster of “general function prediction only” was the largest group (20.23%; 2,885 unigenes), followed by “replication, recombination, and repair” (8.28%; 1,181 unigenes) and “post-translational modification, protein turnover, chaperones” (7.93%; 1,131 unigenes).

### Enrichment analysis of differentially-expressed genes

**Figure 5:**
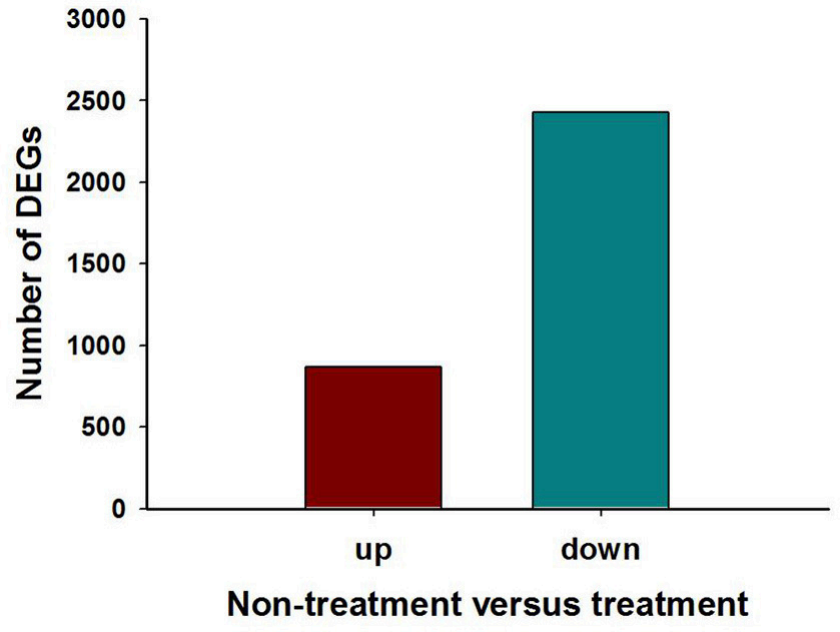
**Differential gene expression analysis between *Harmonia axyridis* adults that were (or were not) exposed to a sublethal dose of beta-cypermethrin**. The number of differentially-expressed genes (up-regulated and down-regulated) in the gene libraries of exposed or un-exposed ladybirds is summarized.

**Figure 6:**
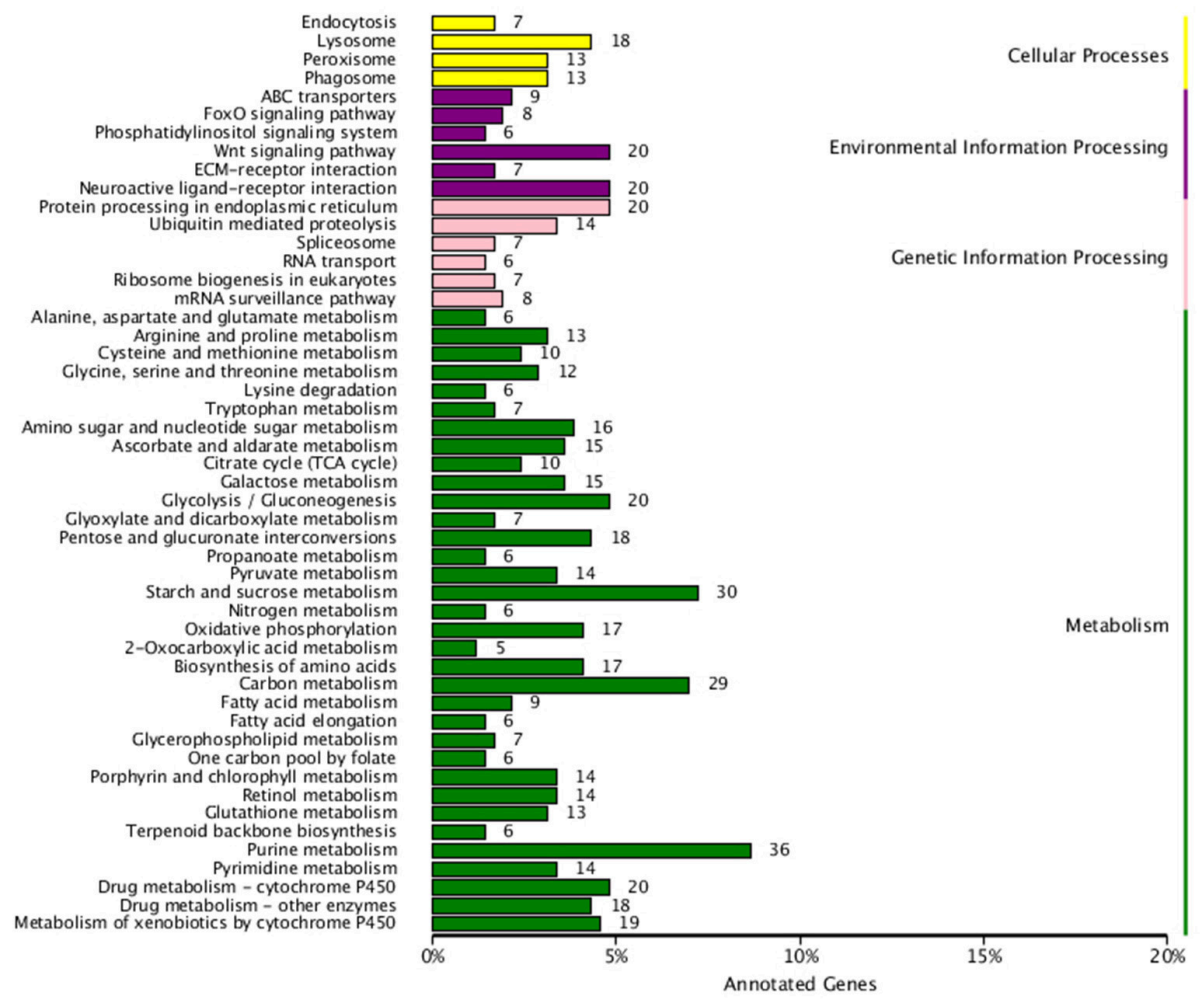
**Kyoto Encyclopedia of Genes and Genomics (KEGG) pathway enrichment of differentially-expressed genes of *H. axyridis* after being exposed to a sublethal dose of beta-cypermethrin**. X axis is the number of DEGs clusted in KEGG pathway. Y axis is the KEGG pathway.

**Figure 7:**
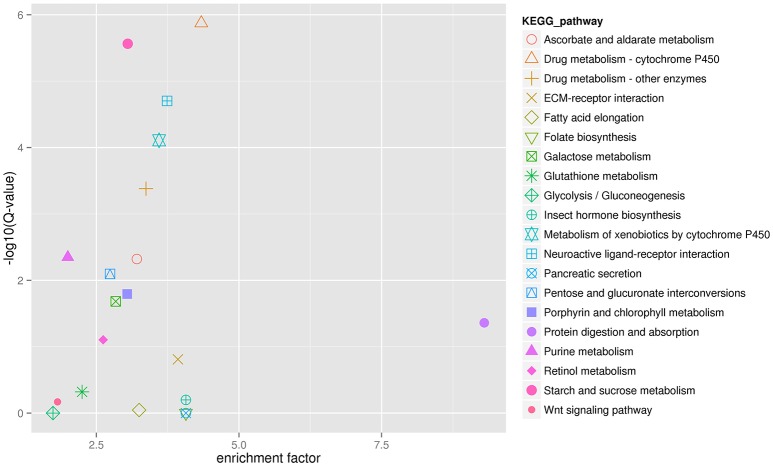
**The 20 most enriched KEGG pathways of *H. axyridis* after being exposed to a sublethal dose of beta-cypermethrin. X axis is enrichement factor**. Y axis is the log_10_(*Q*-value). Each symbol represent an KEGG pathway. The name of KEGG pathway were showed in right legend.

The analysis showed that exposing *H. axyridis* adults to a sublethal dose of beta-cypermethrin had a significant effect on their transcriptome profile. Most of the differentially-expressed genes were down-regulated (Figure 5). For KEGG enrichment analysis, a total of 415 unigenes were assigned to 103 KEGG pathways, mainly involving metabolism, genetic information processing, environmental information processing, and cellular processes. Among these pathways, 11 pathways were significantly enriched with *Q*-values < 0.05 (Figure 6). Drug metabolism, starch and sucrose metabolism and neuroactive ligand-receptor interaction were the major enrichment pathways (Figure 7). Among these pathways, two esterase and four glutathione S-transferases were identified. And all of these genes were down-regulation when *H. axyridis* confronted with beta-cypermethrin after 24 h (Table 1). Taken together, this enrichment analysis indicated that drug metabolic and energy pathways played vital roles in the *H. axyridis* response to a sublethal dose of beta-cypermethrin.

**Table 1:** **Detoxify-related genes differentially transcribed in *Harmonia axyridis* adult following treated by sublethal doses of beta-cypermethrin**.

**Gene style**	**Gene ID**	**Gene name**	**Fold**	**FDR**
Esterases	c16639.graph_c0	PREDICTED: cholinesterase 1-like [*Tribolium castaneum*]	–5.22	1.33E-07
	c32474.graph_c0	Esterase [*Leptinotarsa decemlineata*]	–4.38	8.43E-09
Glutathione S-transferase	c11406.graph_c0	glutathione S-transferase D2 [*Tribolium castaneum*]	–3.39	0.001382
	c26095.graph_c0	PREDICTED: glutathione S-transferase 1 [*Tribolium castaneum*]	–5.60	3.82E-12
	c29610.graph_c0	PREDICTED: microsomal glutathione S-transferase 1 [*Tribolium castaneum*]	–2.70	0.001169
	c29943.graph_c0	PREDICTED: glutathione S-transferase 1 [*Bactrocera cucurbitae*]	–6.43	3.03E-14

## Discussion

The sublethal effect of chemical pesticides is regarded as an additional pressure to arthropods in agroecosystems (Desneux et al., 2007; Guedes et al., 2016). Although this sublethal toxic environment may not fatally threaten the insects, it may influence their biology, behavior, and physiological performance. Exploring the response mechanisms of the insects to sublethal doses of pesticides, especially in the insect predators or parasitoids, may help us to develop additional techniques to protect these “biocontrol agents” from toxicological damage of pesticides (Moscardini et al., 2013).

In this study, we first evaluated the side-effects of a sublethal dose of beta-cypermethrin on the flight, and locomotion performances and respiration of *H. axyridis*. These features are related to population migration and foraging behavior (e.g., for prey) in the predacious ladybird *H. axyridis*. Moreover, we analyzed the transcriptional responses of *H. axyridis* that could be associated to changes in flight and locomotion performances, and in respiration, resulting from exposure to a sublethal dose of beta-cypermethrin. These findings may help us to better understand the adaptation of predatory ladybirds to sublethal doses of insecticides, and may enable improving the use of ladybirds in IPM systems.

### Changes in flight, locomotion performances, and respiration metabolism in response to beta-cypermethrin

Flight and locomotion performances are very important for predatory ladybirds. These features are associated with prey hunting. They are also related to migration, colonization, and dispersion in agroecosystems. These features are of great importance when ladybirds face poor environments, such as food shortages, or in high intraspecific density (Zera, 1997). In the present study, we found that exposure to a sublethal dose of beta-cypermethrin induced succinic female adults of *H. axyridis* to initiate flight more often, and fly for longer times and distances with increased speed. In addition, *H. axyridis* exposed to beta-cypermethrin showed significantly higher walking frequency and duration, longer walking distance, and higher speed. Pesticides residues may thus stimulate a general motion behavior that includes walking and flying. Sublethal doses of pesticides can impact flight and locomotion of various insects both negatively or positively, depending on the species. The parasitoid wasp *Encyrtus saliens* increased its walking speed after being exposed to a sublethal dose of malathion (Linn and Roelofs, 1985). The cabbage looper moth *Trichoplusia ni* presented hyperreflexia after being exposed to a sublethal dose of pesticides and initiated flight more frequently (Hoy and Dahlsten, 1984). By contrast, the green peach aphid *M. persicae* reduced its willingness to fly after being exposed to a sublethal dose of aldicarb (Boiteau et al., 1985). Sometimes the sublethal effects of pesticides on the activities of insects are indirect. There are numerous studies showing how the flight of the pink bollworm moth *Pectinophora gossypiella* is influenced by sublethal doses of neurotoxic insecticides (Haynes, 1988). Wiles and Jepson (1994) found that residues of deltamethrin have sublethal effects on predatory ladybirds. Seven spots ladybirds were driven out of wheat agroecosystem containing deltamethrin and migrated to wheat land lacking pesticide. There are two possible explanations for ladybirds to migrate out of agroecosystems containing residual pesticides. On the one hand, the ladybirds may follow the migration of their prey out from the contaminated ecosystem. On the other hand, pesticides may have repellent effects on predators (Desneux et al., 2007). Despite the decrease of the prey densities after the application of pesticides, the acclimation of the ladybird to sublethal doses of pesticides may play also a key role for successful IPM programs.

Any insect activity is related to respiratory and energy metabolism, and is influenced by environmental changes (Wong et al., 2016). The flight of insects is associated with the release of hormones and with the metabolism of lipids, fatty acids, or glycerol (Haunerland, 1997; van der Hourst, 2003). We could not find any reports showing how a sublethal dose of pesticides may alter the energy metabolism benefiting flight and locomotion. Locomotion and flight changes triggered by environmental toxicants could be regarded as external performance modifiers, following responsive physiological paths and complex changes of gene expression. Exploring gene expression changes in response to sublethal doses of pesticides may help us to better understand the physiological responses of the predators to the residual pesticides and to reveal the mechanisms of the toxicant-driven acclimation of ladybirds.

### Transcriptional responses to insecticide

The advent of next-generation sequencing has allowed for rapid sequencing and transcriptomic research. A total of 66,506 unigenes were generated in our study. In addition, the BLASTx analysis of Genbank showed that 39.99% of the unigenes have significant homology to functional genes that encode specific proteins. In consequence, 60.01% of the unigenes had no clear homology to known genes. This low percentage of annotated genes was most likely attributed to the absence of specific information on the genome of *H. axyridis*. Due to this absence of information, some *H. axyridis* transcripts, derived from the untranslated regions or from non-conserved domains cannot be annotated.

The red flour beetle, *T. castaneum* is the only beetle species with a completely sequenced genome (Richards et al., 2008). As expected, *T. castaneum* is the species that returned most of the BLAST hits (37.44%) against *H. axyridis* transcripts. Transcriptome sequences were used to carry out research dealing with the effect of insecticides and diet shifts on ladybirds in a previous study (Zhang et al., 2012; Tang et al., 2014; Li et al., 2016). The new transcriptome data of *H. axyridis* reported here greatly enriches the available genetic information of this ladybird species.

When confronted with insecticides, the detoxification systems of insect are activated to cope with toxic chemicals. Our results demonstrate that a stress through exposure to a sublethal dose of beta-cypermethrin resulted in large variation in the transcriptome profile of ladybirds. Several detoxification pathways were significantly changed. Among these pathways, we detected two esterases and four glutathione S-transferases that were down-regulated. There are several detoxification mechanisms in insects: Cytochrome P450s, glutathione S-transferases, and esterases are recognized as three major detoxifying enzyme systems that are involved in detoxification of various classes of chemicals (Kristensen, 2005; Feyereisen, 2012). Our results suggest that *H. axyridis* recover from insecticide exposure by eliminating toxic chemicals through detoxifying enzymes. The down-regulation of glutathione S-transferases and esterases could play an important role in this process.

Toxic stress (both from direct spraying of chemical agricultural pesticides and from their residues) may be a strong drive for adaptive evolution in arthropods, which may change their population structures, age, and sexual ratios (Desneux et al., 2007; Guedes et al., 2016). In addition, as shown here, some predators in high trophic levels of the food web could show acclimation by increasing flight or locomotion performances, while also changing their development and reproduction strategies as has been reported elsewhere (Xiao et al., 2016). Transcriptional analysis demonstrated that this kind of plasticity of adaptation may result from the selective expression of functional genes related to energy metabolism and detoxification systems. During adaptive evolution, sublethal impacts of environmental stressors may lead to directional selection, by selecting for the genes that may benefit the population subjected to those specific stressors (Schlichting and Pigliucci, 1998; Li et al., 2014). We must rely on the novel differentially-expressed genes found in this study to explore the possible adaptive evolution of predatory ladybirds facing sublethal doses of toxicants.

## Conclusion

In summary, we investigated the sublethal effects of beta-cypermethrin on locomotion, respiration, and detoxifying mechanisms of *H. axyridis*. Our results showed that *H. axyridis* increase their flight, locomotion performances, and respiratory metabolism after being exposed to beta-cypermethrin. In addition, the down-regulation of genes related to detoxification enzyme (glutathione S-transferases and esterases) could play an important role in the detoxifying process in *H. axyridis* when experiencing exposure to pesticides.

## Author contributions

DX, XT, FZ, and SW designed the study; DX, XT, and WW proceeded the experiments; SW and ND analyzed the results; DX, XT, FZ, and SW wrote the manuscript.

### Conflict of interest statement

The authors declare that the research was conducted in the absence of any commercial or financial relationships that could be construed as a potential conflict of interest. The reviewer RL and handling Editor declared their shared affiliation, and the handling Editor states that the process nevertheless met the standards of a fair and objective review.

## Supplementary material

Figure S1 **Length distribution of the unigenes in the assembled *Harmonia axyridis* transcriptome**. The x and y axis represent the length of unigenes and the number of unigenes of the corresponding length, respectively.

Figure S2 **E-value (A)** and species distribution **(B)** of the top BLASTx hits. The BLASTx search was performed against NCBI non-redundant protein database with an E-value cut-off of 10^−5^.

Figure S3 **Gene ontology classification of *Harmonia axyridis* unigenes after BLASTx serach**. Histogram presentation of the gene ontology annotation was generated using the WEGO software. A total of 12,088 unigenes were assigned to the second level to three gene ontology categories: Biological process, cellular component, and molecular function. The y axis represents the percentage of a certain gene ontology term within each category. One unigene could be assigned to more than one gene ontology category.

Figure S4 **Clusters of orthologous groups classification of *Harmonia axyridis* unigenes after BLASTx search**. A total of 14,258 proteins were aligned to the COG protein database, and classified functionally into 25 classes. Each functional class is denoted by capital letters in the x axis. The y axis represents the number of unigenes in the corresponding functional class.
