# Supplementary figures and images for: Modification of Flight and Locomotion Performances, Respiratory Metabolism, and Transcriptome Expression in the Lady Beetle Harmonia axyridis through Sublethal Pesticide Exposure
Source: Front Physiol. 2017 Feb 10;8:33. doi: 10.3389/fphys.2017.00033 (PMC5300995; doi:10.3389/fphys.2017.00033)

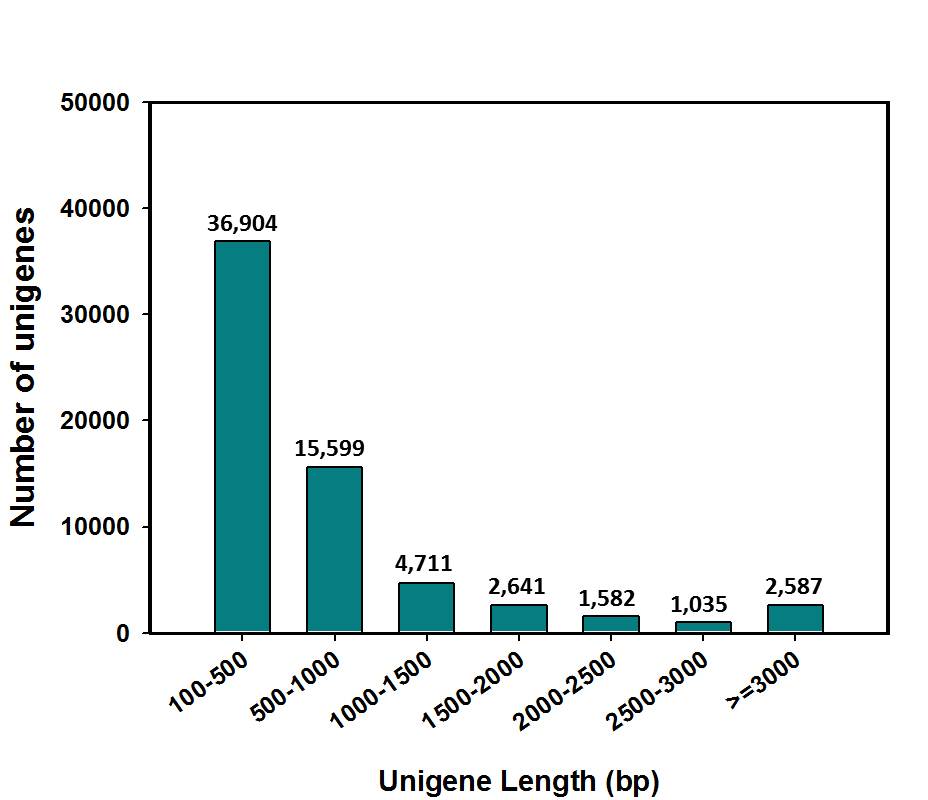

Supplement: Figure S1 — Length distribution of the unigenes in the assembled Harmonia axyridis transcriptome. The x and y axis represent the length of unigenes and the number of unigenes of the corresponding length, respectively. [file Image1.JPEG]

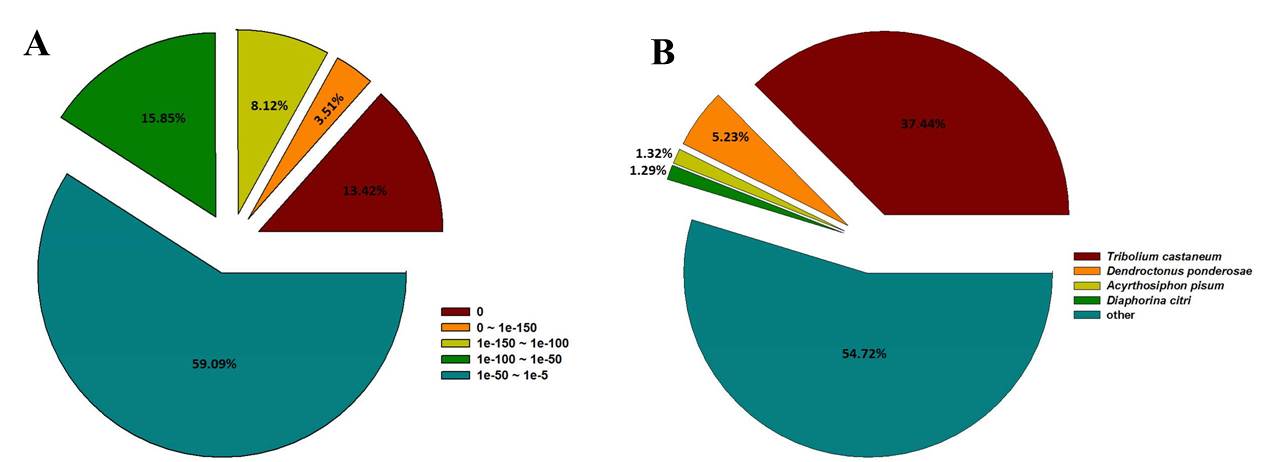

Supplement: Figure S2 — E-value (A) and species distribution (B) of the top BLASTx hits. The BLASTx search was performed against NCBI non-redundant protein database with an E-value cut-off of 10−5. [file Image2.JPEG]

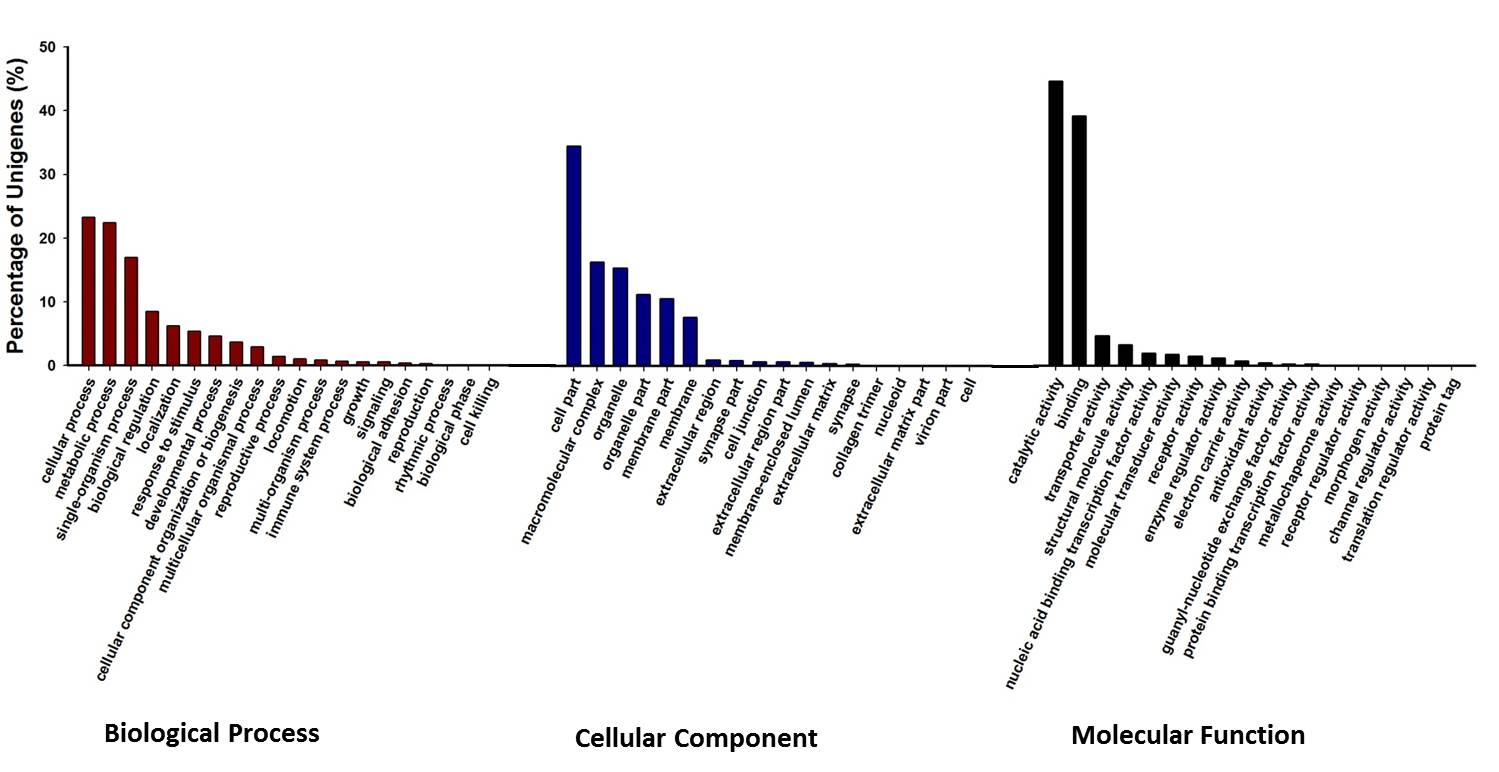

Supplement: Figure S3 — Gene ontology classification of Harmonia axyridis unigenes after BLASTx serach. Histogram presentation of the gene ontology annotation was generated using the WEGO software. A total of 12,088 unigenes were assigned to the second level to three gene ontology categories: Biological process, cellular component, and molecular function. The y axis represents the percentage of a certain gene ontology term within each category. One unigene could be assigned to more than one gene ontology category. [file Image3.JPEG]

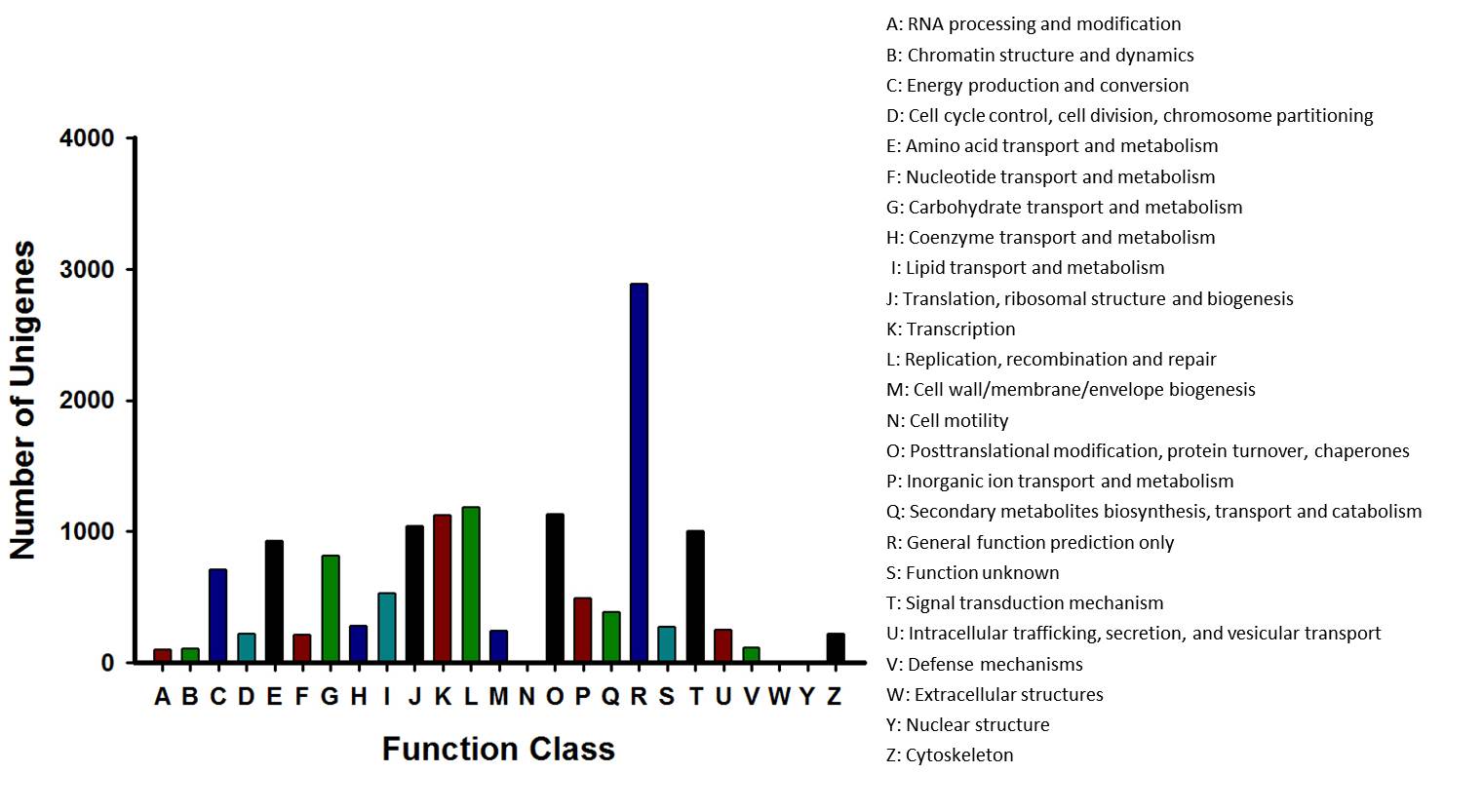

Supplement: Figure S4 — Clusters of orthologous groups classification of Harmonia axyridis unigenes after BLASTx search. A total of 14,258 proteins were aligned to the COG protein database, and classified functionally into 25 classes. Each functional class is denoted by capital letters in the x axis. The y axis represents the number of unigenes in the corresponding functional class. [file Image4.JPEG]
